# 12-Month Trajectories of Health-Related Quality of Life Following Hospitalization in German Cancer Centers—A Secondary Data Analysis

**DOI:** 10.3390/curroncol31050177

**Published:** 2024-04-23

**Authors:** Martin Eichler, Klaus Hönig, Corinna Bergelt, Hermann Faller, Imad Maatouk, Beate Hornemann, Barbara Stein, Martin Teufel, Ute Goerling, Yesim Erim, Franziska Geiser, Alexander Niecke, Bianca Senf, Joachim Weis

**Affiliations:** 1 Medical Faculty, National Center for Tumor Diseases (NCT/UCC), Technical University Dresden, 01307 Dresden, Germany; beate.hornemann@ukdd.de; 2 Department of Psychosomatic Medicine and Psychotherapy, Comprehensive Cancer Center Ulm (CCCU), Ulm University Hospital, 89070 Ulm, Germany; klaus.hoenig@uniklinik-ulm.de; 3 Hamburg Hubertus Wald—University Cancer Center (CCC), University Clinic Centre, 20251 Hamburg, Germany; bergelt@uke.de; 4 Department Medical Psychology and Psychotherapy, Comprehensive Cancer Center Mainfranken, University of Würzburg, 97080 Würzburg, Germany; h.faller@uni-wuerzburg.de; 5 Department Internal Medicine and Psychosomatics, University Clinic Centre Heidelberg, 69120 Heidelberg, Germany; maatouk_i@ukw.de; 6 Section of Psychosomatic Medicine, Psychotherapy and Psychooncology, Department of Internal Medicine II, Julius-Maximilian University Würzburg, 97080 Würzburg, Germany; 7 Department of Psychosomatic Medicine and Psychotherapy, General Hospital Nuremberg, Paracelsus Medical University, 90419 Nuremberg, Germany; barbara.stein@klinikum-nuernberg.de; 8 Department Psychosomatic Medicine and Psychotherapy, Comprehensive Cancer Center Essen (WTZ) and LVR University Hospital, University of Duisburg-Essen, 45122 Essen, Germany; martin.teufel@lvr.de; 9 Charité Universitätsmedizin Berlin, Corporate Member of Freie Universität Berlin, Humboldt-Universität and Berlin Institute of Health, 10117 Berlin, Germany; ute.goerling@charite.de; 10 Department Psychosomatic Medicine, University Clinic Centre Erlangen, 91054 Erlangen, Germany; yesim.erim@uk-erlangen.de; 11 Department Psychosomatic Medicine, University Clinic Centre Bonn, 53127 Bonn, Germany; franziska.geiser@ukbonn.de; 12 Faculty of Medicine & University Hospital, Department Psychosomatics and Psychotherapy, University of Cologne, 50937 Köln, Germany; alexander.niecke@uk-koeln.de; 13 University Cancer Center, University Clinic Centre Frankfurt, 60590 Frankfurt, Germany; bianca.senf@eh-darmstadt.de; 14 Protestant College of Darmstadt, University of Applied Sciences, 64293 Darmstadt, Germany; 15 Faculty of Medicine and Medical Center University of Freiburg, Department Self-Help Research, Comprehensive Cancer Center, 79106 Freiburg, Germany; joachim.weis@uniklinik-freiburg.de

**Keywords:** health literacy, health-related quality of life, prospective longitudinal study, SF12

## Abstract

Patient-reported outcomes (PROs) offer a diverse array of potential applications within medical research and clinical practice. In comparative research, they can serve as tools for delineating the trajectories of health-related quality of life (HRQoL) across various cancer types. We undertook a secondary data analysis of a cohort of 1498 hospitalized cancer patients from 13 German cancer centers. We assessed the Physical and Mental Component Scores (PCS and MCS) of the 12-Item Short-Form Health Survey at baseline (t0), 6 (t1), and 12 months (t2), using multivariable generalized linear regression models. At baseline, the mean PCS and MCS values for all cancer patients were 37.1 and 44.3 points, respectively. We observed a significant improvement in PCS at t2 and in MCS at t1. The most substantial and significant improvements were noted among patients with gynecological cancers. We found a number of significant differences between cancer types at baseline, t1, and t2, with skin cancer patients performing best across all time points and lung cancer patients performing the worst. MCS trajectories showed less pronounced changes and differences between cancer types. Comparative analyses of HRQoL scores across different cancer types may serve as a valuable tool for enhancing health literacy, both among the general public and among cancer patients themselves.

## 1. Introduction

Over the past five decades, patient-reported outcomes (PROs) related to health-related quality of life (HRQoL) have undergone a substantial evolution, witnessing a notable expansion in their application across various domains. They now serve a diverse array of purposes within medical research and clinical practice. Beyond their conventional role in assessing medical outcomes in observational studies and clinical trials, PROs are increasingly utilized as prognostic or diagnostic instruments and are therefore progressively integrated into routine clinical practice and clinical quality management protocols.

PROs are commonly defined as patients’ self-reports regarding their health status, devoid of interpretation or alteration by a third party [1]. While the resulting measure (outcome) indeed originates directly from the patient, the interpretation of PRO measures necessitates the use of contextualization parameters. These parameters, such as thresholds for clinical relevance, minimal clinically important differences, and reference values, facilitate the meaningful interpretation of PRO data. As PROs are typically gathered through standardized questionnaires, known as patient-reported outcome measurement (PROM) tools, their outcomes are predetermined by the structure of the measurement instrument (i.e., the questions and scales employed), rather than being conveyed in the patient’s own language or expression.

PROs serve as a structured and standardized adjunct to, or enhancement of, clinical encounters like physician interviews. They facilitate streamlined documentation and enhance comparability among patients and across different time points. Available in paper or electronic formats, PROs enable the dissection of the doctor–patient dialogue both spatially and temporally. However, it is essential to acknowledge that, unlike individual consultations, PROMs are inherently generic and thus cannot entirely replace the physician interview.

In cancer research, the generic nature of PROMs has spurred the development of increasingly nuanced entity-, site-, or therapy-specific PROMs. Additionally, the utilization of item libraries, containing pre-evaluated individual PRO items curated for specific medical contexts, has gained traction. Key organizations such as the American Functional Assessment of Chronic Illness Therapy (FACIT) and the European Organization for Research and Treatment of Cancer (EORTC) have been instrumental in advancing this domain [2,3].

The generic format of PROM-results presents opportunities, particularly within the realms of informed policy-making and health literacy. For patients, they serve as a valuable tool for comprehending their well-being within the broader population context or within their specific (disease) population. Moreover, for the general populace and health policy makers, PROMs can aid in identifying vulnerable cohorts and gaps in care provision, akin to the role played by cancer registry reporting in tracking incidence and mortality rates.

Although there are many longitudinal studies for specific singular types of cancer and/or treatment situations to date, only a limited number of studies provide such insights across various cancer types, let alone comprehensive comparisons that encompass the full spectrum of malignant diseases. In an unsystematic review of the literature, we found no longitudinal investigations comparing different cancer types. This is probably not surprising, as specific clinical interest often focuses on individual precisely delineated situations, rather than overview comparisons, which are also susceptive to systematic bias due to the heterogenous patient population. Findings from cross-sectional analyses have been inconclusive. An unadjusted analysis assessed by the EORTC C30 sum-score conducted on a cohort of 4020 patients spanning 11 cancer types [4] observed that individuals with mesothelial and soft-tissue cancers experienced the most pronounced restrictions in HRQoL, followed by patients afflicted with respiratory tract and female genital organ malignancies. Another study focusing on advanced cancer patients, utilizing the EQ5D and the FACT-G instruments, identified disparities between cancer types across various outcomes; however, consistent patterns in these differences were lacking [5]. A third investigation failed to detect significant differences in HRQoL outcomes among survivors of colon, breast, cervix, and thyroid cancers [6].

This study aimed to investigate the following research questions:

(1) How do HRQoL trajectories vary among distinct cancer types up to 12 months post-hospitalization? Are there discernible differences in baseline scores and identifiable trajectories across different cancer types?
(2) How does the HRQoL, both in terms of mental and physical components, of individuals with different types of cancer compare to that of the general population?

## 2. Materials and Methods

We conducted a secondary analysis of longitudinal data derived from a prospective observational study conducted in across 13 certified German Comprehensive Cancer Centers (CCCs), which adhere to the German standard for certified Oncology Centers of Excellence. The study was registered in the German Clinical Trials Registry under the identifier DRKS00004860. Recruitment took place within 2–3 organ tumor centers, each self-selected by collaborators at every CCC, spanning a period of up to nine months, from 2014 to 2015. Variables were systematically assessed at baseline (t1), which occurred during hospitalization, along with follow-up assessments at 6 months (t2) and 12 months (t3) post-baseline. For the t2 and t3 assessments, questionnaires were dispatched via mail to the patients’ home addresses, accompanied by a pre-paid return envelope. For non-responders, a maximum of two reminders were issued. In the pre-test, the time needed for completing the HRQoL questionnaire was about 10 min. A more comprehensive description of the study methodology was previously published [7].

Eligible participants included individuals aged 18 years or older; diagnosed with cancer at any site, regardless of prognosis, stage of disease, or time since diagnosis; and possessing sufficient proficiency in the German language. Patients with acute and severe psychiatric disorders or significant cognitive impairment, hindering their understanding of the questionnaires, were excluded. Recruitment took place during hospitalization for oncological therapy at one of the participating study centers, with participation contingent upon providing written informed consent. Approval for the study was obtained from the ethics committees of the University of Freiburg (No. 139/13) and the respective participating centers. For the current analysis, only patients who provided HRQoL data at baseline were included.

HRQoL was assessed using the 12-Item Short-Form Health Survey (SF-12), which provides two subscales: a Physical Component Score (PCS) and a Mental Component Score (MCS) [8]. The PCS encompasses domains related to physical functioning, role physical, bodily pain, and general health, while the MCS comprises vitality, social functioning, role emotional, and mental health. Both PCS and MCS values were calculated using norm-based methods, with norms derived from the German general population [9]. In this population, both scales have a mean of 50 and a standard deviation of 10. Scores above 50 indicate values higher than the average, while scores below 50 indicate values below the average. Sociodemographic variables were collected via self-report, using a standardized form, while medical data were extracted from participants’ medical records.

To describe the study population, we evaluated the model variables as follows:

- For age and time since diagnosis, we calculated the median and the interquartile range (IQR).
- Categorical variables were presented as absolute numbers and relative frequencies.
- Variables were stratified by cancer type.

Additionally, to assess potential bias due to dropouts during follow-up, we evaluated dropouts stratified by cancer type. An age and sex standardized comparison was performed using reference values from the healthy German population [10].

PCS and MCS were analyzed across three time points, using generalized linear regression models with an undefined covariance structure. The independent variable was cancer type, categorized as follows: mamma (C50), skin (V34, C35), gynecological (C51–C58), digestive tract (C16–C21), lung (C34), oral/facial (C01–C14), prostate/germ cell (C61, C62), and all other cancer types.

To control for potential confounders, the following baseline variables were included: age at diagnosis; sex (male or female); time of survey (0, 6, or 12 months); time since diagnosis (up to 1 month, 2–4 months, 5–12 months, more than 12 months, or unknown); metastatic disease; T-stage (T1, T2–T4, or Tx); N-stage (0, 1–3, or unknown); disease status (complete remission, partial remission/stable, or progression, unknown); reason for hospitalization at baseline (primary cancer, second cancer, recurrence, or unknown); physical comorbidities (yes/no); emotional comorbidities (yes/no); treatments performed at baseline (radiotherapy (no, treated, or in treatment), chemotherapy (no, treated, or in treatment), or surgery (yes/no)); education (secondary school up to 9 years, secondary school 10 years, vocational baccalaureate, baccalaureate, or unknown); partnership (yes/no); and occupation (blue collar, white collar, civil servant, self-employed, or unknown).

Additionally, interaction terms for time of survey by cancer type and time since diagnosis by cancer type were included. Unstandardized regression coefficients (Bs), 95% confidence intervals (95% CIs), and *p*-values were evaluated. The adjusted means of individual cancer types were plotted graphically across t1, t2, and t3. Statistical analyses were conducted using SPSS V.27 (IBM Corporation, Armonk, New York, NY, USA). ChatGPT Modell 4.0 was used for spelling and grammar checks.

## 3. Results

### 3.1. Sample Description

During the recruitment period, a total of N = 6088 patients were treated in the recruiting cancer centers. Of these, N = 3046 patients (50.0%) were approached for participation, and among those, N = 1741 patients (57.2%) agreed to participate. Dropout rates and reasons are detailed elsewhere [7]. Due to missing data in the outcome and covariates, a total of 1498 patients were included in the analysis (Table 1). The mean age of the analyzed participants was 59 years (interquartile range: 52; 66). Among the participants, N = 871 (58.1%) were women (Appendix A Table A1). The most prevalent cancer types included breast cancer (21.4%), skin cancers (15.1%), gynecological cancers (14.0%), and cancers of the digestive tract (13.5%) (Appendix A Table A1).

### 3.2. Longitudinal Dropout Analysis

For the analysis of PCS, data were available for 1498 patients at baseline, 934 patients (62.3%) at 6 months, and 845 patients (56.4%) at 12 months. Similarly, for MCS, data were available for 1498 patients at baseline, 935 patients (62.4%) at 6 months, and 848 patients (56.6%) at 12 months. The highest dropout rate was observed among lung cancer patients, with data available for only 37.6% of patients at t2. In contrast, the lowest dropout rate was observed among prostate/germ cell cancer patients, with data available for 79.8% of patients at t2 (Table 1).

### 3.3. Baseline Quality of Life—Comparison with Norm Population

Age- and gender-adjusted PCS was 37.1 points (SD 9.0) for all cancer patients at baseline (men, 37.3 (SD 9.1); women, 37.1 (SD 9.0)). The difference from the norm population was 12.9 points, which fell below one standard deviation (10 points) of the norm population mean.

**Figure 1:**
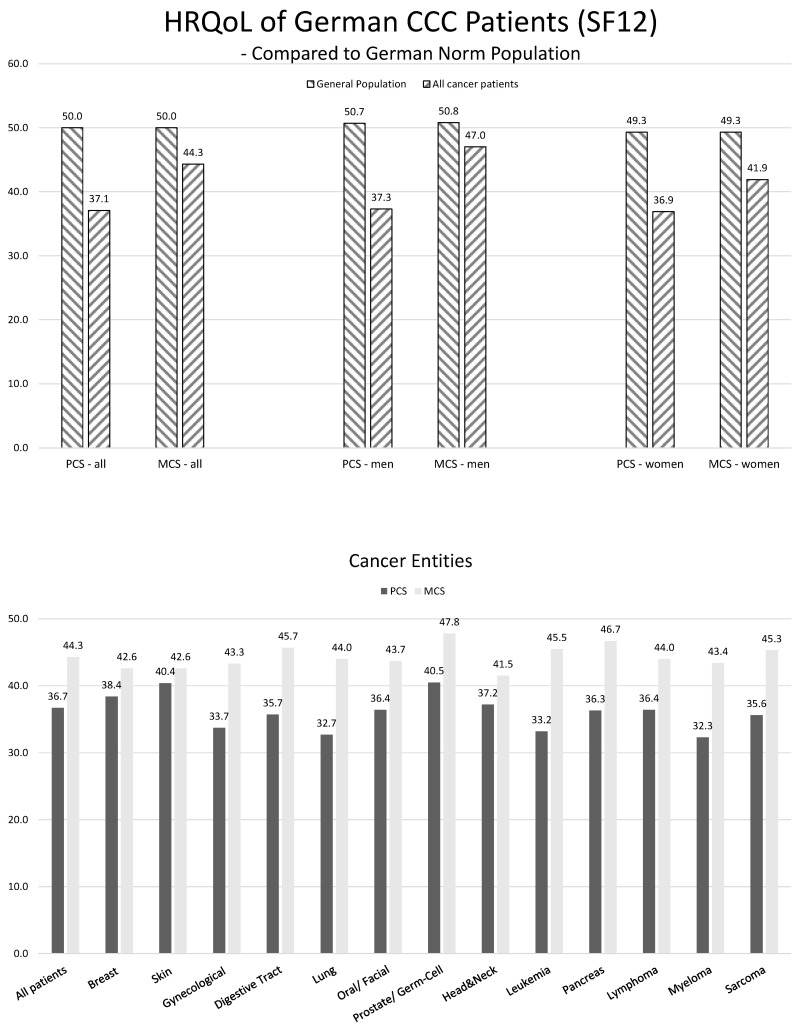
Top row: age and gender standardized health-related quality of life among patients of German Comprehensive Cancer Centers at the time of hospitalization—a comparison with normative data from the general German population (Wirtz, 2019). Bottom row: unstandardized comparison of health-related quality of life among selected cancer types. CCC = Comprehensive Cancer Center; PCS = SF12 Physical Health Summary Scale; MCS = SF12 Mental Health Summary Scale.

MCS was 44.3 points (SD 11.1) at baseline (men, 47.0 (SD 10.5); women, 41.9 (SD 11.2)). The difference from the norm population was 5.7 points, which was within one standard deviation of the norm population mean (Figure 1).

In the unadjusted comparison of different cancer types, myeloma patients had the lowest PCS values (32.3, SD 8.5), followed by lung cancer patients (32.7, SD 8.2). Conversely, prostate/germ-cell cancer patients (40.5, SD 8.6) and those with skin cancer (40.4, SD 8.4) demonstrated the highest PCS values.

In terms of MCS values, patients with cancers of the head and neck exhibited the lowest scores (41.5, SD 11.5), while those with prostate/germ-cell cancer had the highest (47.8, SD 11.0) (Figure 1).

### 3.4. Generalized Linear Model—General Results

PCS values were 36.7 at t1, 39.2 at t2, and 40.6 at t3. In the multivariable generalized linear model, we observed an improvement in PCS after 12 months (B = 2.2, 95% CI 1.2–3.2) for all cancer patients. Lung cancer patients (B = −4.2, 95% CI −8.3; −0.2) and patients with gynecological cancers (B = −2.6, 95% CI −5.3; −0.01) had significantly worse PCS values compared to breast cancer patients, who served as reference.

MCS values were 44.3 at t1, 45.9 at t2, and 46.6 at t3. In the multivariate model, we observed an improvement in MCS after 6 months (B = 2.8, 95% CI 1.6–4.0), and it remained stable after 12 months (B = 2.8, 95% CI 1.6–4.0) (Table 2).

### 3.5. Generalized Linear Model—Comparison of Cancer Types over Time

**Figure 2:**
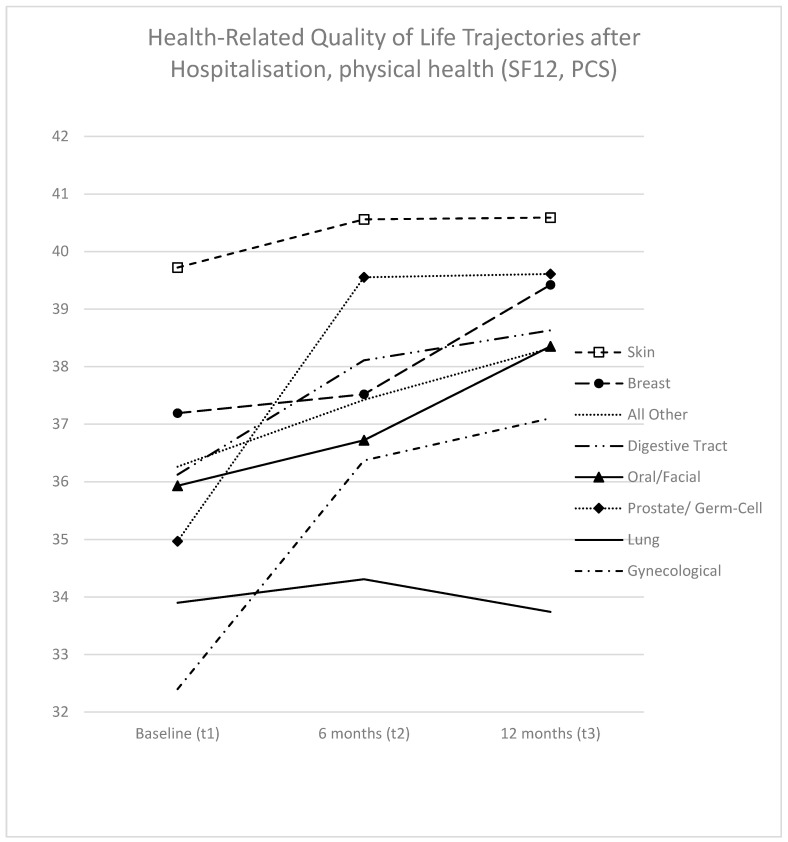
Trajectories of HRQoL scores (PCS) following hospitalization at baseline: adjusted values for age at diagnosis, sex, time of survey, time since diagnosis, metastatic disease, T-stage, N-stage, disease status, reason for hospitalization, physical comorbidities, emotional comorbidities, radiotherapy, chemotherapy, surgery, education, partnership, and occupation. Interactions considered: time of survey by cancer type and time since diagnosis by cancer type. PCS = SF12 Physical Health Summary Scale.

As depicted in Figure 2, among the seven cancer types analyzed, six exhibited improvements in PCS over the study period. Notable improvements were observed in gynecological cancer patients (4.7 points, statistically significant) and prostate/germ cell cancer patients (4.6 points, not statistically significant). Conversely, lung cancer patients were the sole group demonstrating a slight decrease in PCS over time (−0.2 points), which was not statistically significant.

Adjusted baseline values of the cancer types at baseline exhibited considerable variation, with a range of 7.3 points. Skin cancer patients displayed the highest baseline PCS values (39.7, 95% CI 38.0; 41.5), while gynecological cancer patients exhibited the lowest (32.4, CI 30.5; 34.3). By t2, the PCS values converged to a margin of 6.9 points (or 3.7 points if excluding lung cancer). Skin cancer patients displayed the highest PCS values at t2 (40.6, 95% CI 38.7; 42.5), whereas lung cancer patients had the lowest (33.7, CI 31.2; 36.3) (Supplementary Table S1; Figure 2). In the direct comparison of analyzed individual cancer types, significant differences were observed across all three time points (Supplementary Table S1). Skin cancer patients performed significantly better than four other entities (gynecological, digestive tract, lung, and prostate/germ cell) at baseline, while lung cancer patients performed significantly worse than four other entities (breast, skin, digestive tract, and prostate/germ cell) at t2.

**Figure 3:**
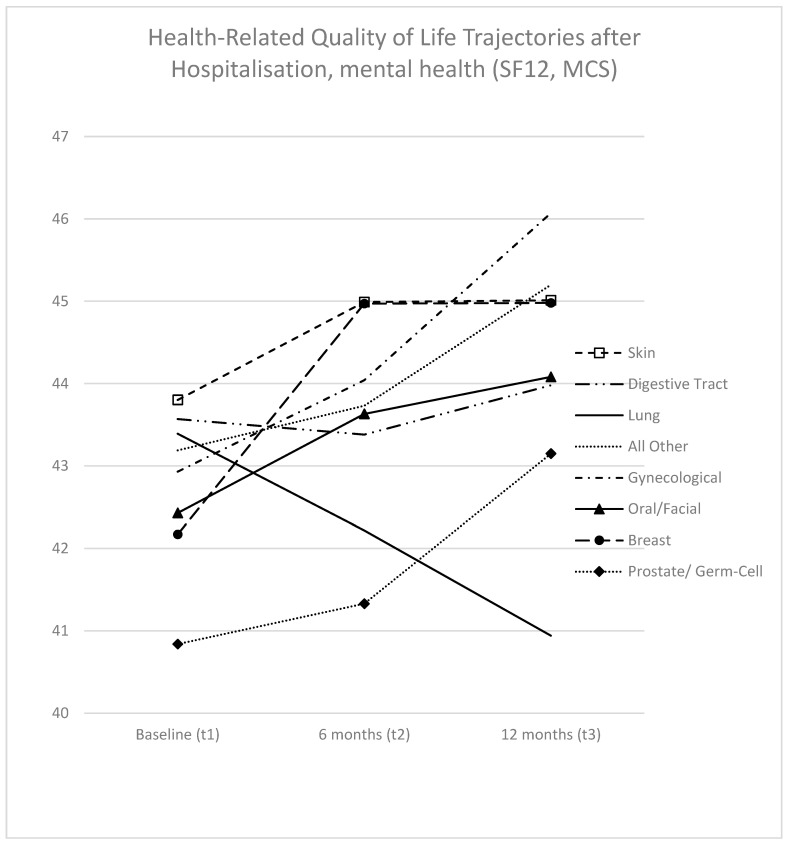
Trajectories of HRQoL scores (MCS) following hospitalization at baseline: adjusted values for age at diagnosis, sex, time of survey, time since diagnosis, metastatic disease, T-stage, N-stage, disease status, reason for hospitalization, physical comorbidities, emotional comorbidities, radiotherapy, chemotherapy, surgery, education, partnership, and occupation. Interactions considered: time of survey by cancer type and time since diagnosis by cancer type. MCS = SF12 Mental Health Summary Scale.

As illustrated in Figure 3, trajectories concerning MCS were somewhat similar but less pronounced. With lung cancer patients as the exception (exhibiting a 2.5-point decrease, which was not statistically significant), all cancer types demonstrated improvements in MCS. The highest improvements were observed in gynecological cancer patients (3.1 points, though not statistically significant) and breast cancer patients (2.8 points, also not statistically significant).

The baseline values of the cancer types at baseline displayed differences within a range of 3.0 points. Skin cancer patients exhibited the highest MCS values (43.8, 95% CI 41.5; 46.1), while prostate/germ cell cancer patients had the lowest (40.8, CI 36.0; 45.7). These differences between groups did not converge at t2 (5.1 points, or 2.9 points if excluding lung cancer). The highest MCS values at t2 were observed for gynecological cancer patients (46.1, 95% CI 43.4; 48.7), while the lowest were seen for lung cancer patients (40.9, CI 37.6; 44.3) (refer to Supplementary Table S2; Figure 3). In the direct comparison of analyzed individual cancer types, we found no significant differences at any time point (Supplementary Table S2).

## 4. Discussion

### 4.1. Results in Context

The PCS values of hospitalized cancer patients were notably lower at baseline, exhibiting a difference of 12.9 points compared to scores of the German norm population. This mean deviates more than one standard deviation from the mean of the norm population. Slightly more than 90% of the German standard population has a higher PCS value than the average value of the patients surveyed. The differences in MCS were less pronounced but still significant, with a gap of 5.7 points. In this case, slightly more than 71.5% of the German standard population has a higher MCS value than the average value of the patients surveyed. These differences in physical and mental functioning are well documented in the literature, e.g., for sarcoma, prostate, and head and neck cancer patients [11,12,13]. The observed gender difference in MCS is also well described in the literature: woman tend to report greater psychological restrictions and psychological distress than men [14,15].

Over the course of 12 months, both PCS and MCS demonstrated improvements of 2.2 and 2.8 points, respectively. Considering the baseline disparities between cancer patients and the norm population in both PCS and MCS, it is noteworthy that PCS showed a slight convergence towards the normal population, while MCS exhibited a more distinct approximation. There are some studies on short-term HRQoL trajectories for patients with various cancer entities. Generally, improvements are anticipated following treatment [16,17,18]. However, medium- or long-term restrictions in the HRQoL of cancer survivors are well-documented in the literature [19,20], showing that improvement is not universal [21].

Baseline values for PCS varied significantly among the analyzed cancer types. For heuristic purposes, it appears feasible to categorize the cancer types at baseline into several groups: a top-performing group (Group 1), comprising skin cancer patients; a moderately performing group (Group 2), including breast, digestive tract, oral/facial, and prostate/germ cell patients; and a bottom-performing group (Group 3), consisting of lung cancer and gynecological cancer patients. Significant differences were observed between Groups 1 and 3. After 12 months, differences between Groups 1 and 2 diminished, leaving lung cancer patients as the sole members of Group 3. In terms of 12-month trajectories, it appears also feasible to form heuristic groups. Trajectory Group 1 exhibited stable trajectories, represented by lung cancer and skin cancer patients. Trajectory Group 2, comprising breast, digestive tract, and oral/facial cancer patients, showed slight (and insignificant) improvements. Trajectory Group 3, including prostate/germ cell and gynecological cancer patients, demonstrated larger (and partly significant) improvements.

Baseline values for MCS exhibited a narrower range compared to PCS, making it challenging to categorize into distinct groups even for heuristic purposes. This difficulty persists after 12 months, with the exception of lung cancer patients, who followed a distinct trajectory compared to all other entities. While MCS for lung cancer patients deteriorated (insignificantly), MCS for all other cancer groups showed slight increases (none being significant).

The ability to contrast our comparative analysis with existing research is limited. No other longitudinal studies were identified, and existing cross-sectional papers utilized different PROMs and had varying inclusion criteria.

The study by Hinz et al., which analyzed cancer patients across all settings (inpatient, outpatient, and rehabilitation), reported some similar PCS cross-sectional results. Notably, skin cancer patients ranked among those with the highest scores in physical functioning, while patients with lung cancer and gynecological cancer were among those with the lowest scores similar to our findings [4]. The study by Pickard et al., which included advanced cancer patients, indicated that lung cancer patients had one of the highest proportions of problems concerning activities of daily life and the lowest means in the overall EQ-5D index, which is in line with our results. However, results from the FACT-G differed, with head and neck cancer patients exhibiting the lowest scores in functional and physical well-being [5].

Upon examining the results of cancer specific studies, the observed limitations in PCS among lung cancer patients appear plausible. Morrison et al. stated that lung cancer is associated with a higher symptom burden compared to other cancers [22]. Hechtner et al., in their comparison of lung cancer patients with the general population, noted large differences in symptoms and functioning, particularly regarding dyspnea, fatigue, and physical function [23]. Similarly, the relatively good performance of skin cancer patients in the PCS aligns with findings from entity-specific studies. Cornish et al. reasoned that, owing to the high proportion of patients undergoing local surgical excision, HRQOL impairment is primarily influenced by psychological factors and, to a lesser extent, by long-term therapy-induced events, as often seen in other cancers [24].

Finding comparative research for MCS proves even more challenging than for PCS. While Hinz et al. reported the highest score for emotional functioning in patients with cancers of the male genital organs [4], our observations placed this group towards the lower end of the spectrum. Several larger studies have presented the prevalence of psychological distress/mental disorder in different groups of cancer patients. These categories of PROMs are notably distinct from the MCS value but at least comparable in regard to some aspects. Linden et al. reported that patients with lung or gynecological cancer exhibited the highest levels of distress at the time of cancer diagnosis, while prostate cancer patients showed the lowest [14]. With respect to the prevalence of any mental disorder, Mehnert et al. found breast cancer patients to be the most affected, followed by patients with head and neck cancer. The lowest prevalence was observed in patients with pancreatic cancer and stomach/esophagus cancers [25]. Zabora et al., in a study that was published already in 2001, reported an overall distress prevalence rate of 35.1%. The rate varied from 43.4% for lung cancer to 29.6% for gynecological cancers [26].

For a comprehensive comparison of HRQoL trajectories across different cancer types throughout the disease course, larger and more complete datasets would be invaluable, especially when including rarer cancer entities. Establishing a comprehensive understanding of HRQoL trajectories is important for informing both the general public and individual cancer patients. With more extensive and comprehensive datasets, conducting more nuanced analyses that capture the heterogeneity of cancer types becomes feasible. Such an analysis could involve further stratification based on factors such as disease severity or time since diagnosis. In our current analysis, we incorporated additional variables primarily as potential confounding factors in the model.

One possible method to create a more robust empirical foundation for this purpose is by merging existing datasets into larger, publicly accessible databases. Project Data Sphere, which is collecting historical clinical trial data in oncology, could serve as a model in this regard [27]. In the realm of HRQoL research, we are aware of at least one ongoing project commissioned by the EORTC Quality of Life group that could provide the necessary data for the analyses described above [28]. This project is dedicated to developing a dynamic IT infrastructure designed to manage and merge data from numerous EORTC- funded research projects that include HRQoL data as outcomes.

Another option is to incorporate the collection of HRQoL data into existing cancer registries. This approach would necessitate either the establishment of new structures for collecting HRQoL data for this purpose—a variety of disease-specific registry projects already collecting PRO data exist [29,30,31]—or the utilization of data collected in routine clinical care, which is increasingly being established in clinics worldwide.

All possible solutions carry a range of challenges. Merging datasets involves significant administrative efforts, and data-protection concerns must be comprehensively addressed. Establishing structures for HRQoL data collection, whether in new or existing registries, is resource-intensive and beset with administrative hurdles [29,31]. Similarly, the routine collection of PROs faces numerous obstacles: logistical and technical barriers must be overcome, data privacy and security must be safeguarded, and the medical staff need to be convinced of the value of the endeavor, among other things [32].

### 4.2. Strengths and Limitations

This paper presents, to our knowledge, one of the few comprehensive analyses of HRQoL trajectories across different cancer types available to date. Supported by a large dataset from all German CCCs at the time, it enabled a broader comparison of various cancer entities. The baseline participation rate of 57% was comparable to that of other studies [11,33], as are the participation rates at follow-up assessments [34].

Dropout rates varied significantly across cancer types, with no reasons for dropout collected. However, higher dropout rates in cancers with elevated mortality rates, especially lung cancer and digestive tract cancers, suggest that death may have been a primary reason for dropout in these instances [35].

Assessing how (different) dropout and participation rates may result in selection bias and its direction is challenging. The study design did not allow for the data collection of medical records after baseline, precluding adjustment for medical interventions or disease status during follow-up. Therefore, it is possible that the observed differences in trajectories are influenced by these variables. To address the heterogeneity of the compared cancer types, we adjusted for a range of potentially relevant variables. However, several potentially relevant factors, such as specific treatment options (e.g., hormone therapies and targeted therapies), could not be adjusted for. In addition, treatment standards have changed in various cancer entities since the survey was conducted.

Since patients were recruited at the time of hospitalization rather than at a fixed point in their disease course, reproducing the results might be challenging. Furthermore, it is important to note that PCS and MCS were designed to measure general HRQoL and not cancer-specific HRQoL. Therefore, certain quality of life-related aspects of cancer may not have been captured by this PROM. The SF-12 was chosen for pragmatic reasons in order to minimize the time required for the patients to complete the survey.

## 5. Conclusions

Hospitalized cancer patients reported considerably worse PCS and MCS values than the German norm population. The differences in PCS values were particularly pronounced. The physical components of HRQoL exhibited variations between cancer types, and over time, the mental components showed significant changes only over time. The majority of groups experienced improvements in short-term HRQoL 12 months after hospitalization, with significant increases observed in gynecological cancer patients. Notably, lung cancer patients appeared to be the most adversely affected group, exhibiting no observed increase in short-term physical HRQoL and a (insignificant) decrease in mental HRQoL.

Comparisons of HRQoL scores across different cancer types could serve as a valuable tool for identifying vulnerable groups and enhancing health literacy in both the general public and cancer patients. However, significant obstacles remain in achieving common and useful standards in this regard.

## Figures and Tables

**Table 1:** Participation at study time points, stratified by cancer type.

Variable		Baseline (PCS) (%)	t2 (PCS) (%)	t3 (PCS) (%)	Baseline (MCS) (%)	t2 (MCS) (%)	t3 (MCS) (%)
All		1741	1741	1741	1741	1741	1741
No HRQoL data (excluded)		224	788	877	224	787	874
Missing covariables (excluded) ^a^		19	19	19	19	19	19
Patients in the model	All	1498 (100)	934 (62.3)	845 (56.4)	1498 (100)	935 (62.4)	848 (56.6)
	Breast (C50)	320 (100)	243 (75.9)	229 (71.6)	320 (100)	243 (75.9)	230 (71.9)
	Skin (C34, C44)	226 (100)	164 (72.6)	149 (65.9)	226 (100)	164 (72.6)	150 (66.4)
	Gynecological (C51–C58)	210 (100)	130 (61.9)	115 (54.8)	210 (100)	130 (61.9)	115 (54.8)
	Digestive tract (C16–C21)	202 (100)	111 (55.0)	97 (48.0)	202 (100)	111 (55.0)	97 (48.0)
	Lung (C34)	125 (100)	55 (44.0)	47 (37.6)	125 (100)	55 (44.0)	47 (37.6)
	Oral/facial (C01–C14)	89 (100)	45 (50.6)	41 (46.1)	89 (100)	46 (51.7)	41 (46.1)
	Prostate/germ cell (C61, C62)	84 (100)	72 (85.7)	67 (79.8)	84 (100)	72 (85.7)	67 (79.8)
	Other/multiple/unknown	242 (100)	114 (47.1)	100 (41.3)	242 (100)	114 (47.1)	101 (41.7)

PCS = SF12 Physical Health Summary Scale; MCS = SF12 Mental Health Summary Scale; HRQoL = health-related quality of life. ^a^ Excluded due to missing age.

**Table 2:** Results of the generalized linear model.

		Model PCS			Model MCS		
Variable	Value	B	95% CI	*p*	B	95% CI	*p*
Time point	Baseline (ref.)						
	t1 (6 month)	0.34	−0.71; 1.39	0.53	2.80	1.58; 4.01	<0.01
	t2 (12 month)	2.23	1.24; 3.23	<0.01	2.81	1.61; 4.01	<0.01
Sex	Female (ref.)						
	Male	0.50	−0.52; 1.52	0.34	2.43	1.10; 3.75	<0.01
Cancer type	Breast (C50) (ref.)						
	Skin (C34, C44)	2.95	−0.12; 6.02	0.06	0.93	−3.49; 5.34	0.68
	Gynecological (C51–C58)	−2.64	−5.26; −0.02	<0.05	1.14	−2.19; 4.48	0.50
	Digestive tract (C16–C21)	0.57	−2.37; 3.51	0.71	1.13	−2.74; 4.99	0.57
	Lung (C34)	−4.21	−8.25; −0.17	0.04	0.61	−5.26; 6.49	0.84
	Oral/facial (C01–C14)	−1.52	−6.95; 3.91	0.58	−4.15	−11.74; 3.44	0.28
	Prostate/germ cell (C61, C62)	2.73	−0.19; 5.65	0.07	3.83	0.25; 7.41	0.04
	Other	0.08	−2.54; 2.70	0.95	3.91	0.31; 7.51	0.03
Age at diagnosis	Per year increase	−0.06	−0.09; −0.03	<0.01	0.11	0.07; 0.15	<0.01
Metastasis until baseline	No (ref.)						
	Yes	−2.31	−3.57; −1.05	<0.01	−0.92	−2.50; 0.66	0.25
	Unknown	0.08	−1.07; 1.24	0.89	0.20	−1.22; 1.61	0.79
N-stage	0 (ref.)						
	1–3	−1.36	−2.47; −0.24	0.02	−1.45	−2.86; −0.03	<0.05
	Unknown	−1.95	−3.29; −0.62	<0.01	−1.50	−3.15; 0.15	0.07
T-stage	T1						
	T2–T4	−0.84	−1.96; 0.28	0.14	−0.81	−2.24; 0.62	0.27
	Tx/unknown	−1.09	−2.51; 0.33	0.13	−0.98	−2.79; 0.82	0.29
Disease status	Complete remission (ref.)						
	Partial remission/stable disease	0.49	−0.83; 1.80	0.47	2.14	0.44; 3.84	0.01
	Progress	−1.77	−3.33; −0.21	0.03	1.17	−0.83; 3.17	0.25
	Unknown	−0.80	−1.96; 0.36	0.18	1.15	−0.36; 2.65	0.13
Disease type	Primary cancer (ref.)						
	Second cancer	−1.58	−3.15; −0.001	<0.05	−1.37	−3.08; 0.35	0.12
	Recurrence	−1.50	−2.64; −0.36	0.01	−0.67	−2.24; 0.91	0.41
	Unknown	0.66	−0.77; 2.08	0.37	1.55	−0.18; 3.28	0.08
Comorbidities—physical	No (ref.)						
	Yes	−1.81	−2.63; −0.98	<0.01	−0.35	−1.39; 0.69	0.51
	Unknown	−1.14	−3.65; 1.37	0.37	−1.76	−5.31; 1.79	0.33
Comorbidities—psychological	No (ref.)						
	Yes	−1.74	−2.86; −0.61	<0.01	−4.34	−5.84; −2.83	<0.01
	Unknown	0.35	−1.50; 2.19	0.71	0.41	−2.17; 2.99	0.75
Radiotherapy	No/unknown (ref.)						
	Treated	−0.90	−2.17; 0.37	0.16	−0.63	−2.14; 0.89	0.42
	In treatment	−0.96	−2.29; 0.36	0.15	−0.63	−2.40; 1.14	0.48
Chemotherapy	No/unknown (ref.)						
	Treated	0.29	−1.01; 1.59	0.66	1.85	0.13; 3.56	0.04
	In treatment	−1.06	−2.15; 0.04	0.06	1.69	0.22; 3.17	0.02
Surgery	No vs. yes	−0.64	−1.63; 0.35	0.20	0.76	−0.48; 1.99	0.23
Time since diagnosis	0/1 month (ref)						
	2–4 months	0.62	−1.52; 2.76	0.57	−0.81	−3.39; 1.77	0.54
	5–12 months	0.57	−2.15; 3.29	0.68	0.01	−3.27; 3.29	1.00
	>12 months	0.91	−2.24; 4.07	0.57	0.50	−3.04; 4.05	0.78
	Unknown	1.10	−2.37; 4.57	0.53	1.52	−1.98; 5.02	0.39
Stable partnership	No (ref.)						
	Yes	−0.01	−0.90; 0.88	0.98	0.67	−0.56; 1.89	0.28
	Unknown	0.84	−1.04; 2.73	0.38	0.16	−2.17; 2.50	0.89
School	Secondary school (up to 9 years) (ref.)						
	Secondary school (10 years)	−0.27	−1.28; 0.75	0.61	1.04	−0.21; 2.28	0.10
	Vocational baccalaureate	−0.02	−1.49; 1.44	0.98	0.01	−1.98; 2.00	0.99
	Baccalaureate	1.01	−0.10; 2.11	0.07	0.75	−0.64; 2.14	0.29
	Unknown	0.07	−1.75; 1.89	0.94	−1.22	−3.92; 1.49	0.38
Work	Blue collar (ref.)						
	White collar	0.70	−0.43; 1.82	0.22	0.84	−0.64; 2.32	0.27
	Civil servant	0.44	−1.18; 2.06	0.60	−0.18	−2.35; 1.99	0.87
	Self-employed	0.17	−1.38; 1.73	0.83	1.49	−0.49; 3.47	0.14
	Unknown	−0.33	−1.67; 1.01	0.63	−0.39	−2.25; 1.47	0.68

PCS = SF12 Physical Health Summary Scale; MCS = SF12 Mental Health Summary Scale; B = non-standardized regression coefficient (indicating a B point increase or decrease in the respective scale); 95% CI, 95% confidence interval; *p* = *p*-value. Interaction terms shown in Supplementary Table S3.

## Data Availability

The data that support the findings of this study are available upon request from the corresponding author. The data are not publicly available due to privacy restrictions.

## Supplementary Materials

The following supporting information can be downloaded at https://www.mdpi.com/article/10.3390/curroncol31050177/s1, Table S1: Trajectories (values) of HRQoL-scores (PCS) after hospitalisation at baseline; Table S2: Trajectories (values) of HRQoL-scores (MCS) after hospitalisation at baseline; Table S3: Results of the Generalized Linear Model.

## Appendix A

**Table A1:** Baseline description stratified by cancer types.

Variable Value	All N = 1498 N (%)/ Median (IQR)	Mamma N = 320 (21.4%) N (%)/ Median (IQR)	Skin N = 226 (15.1%) N (%)/ Median (IQR)	Gynecological N = 210 (14.0%) N (%)/ Median (IQR)	Digestive N = 202 (13.5%) N (%)/ Median (IQR)	Lung N = 125 (8.3%) N (%)/ Median (IQR)	Oral/Facial N = 89 (5.9%) N (%)/ Median (IQR)	Prostate/Germ Cell N = 84 (5.6%) N (%)/ Median (IQR)	All Other ^a^ N = 242 (16.2%) N (%)/ Median (IQR)
Sex Female	871 (58.1)	320 (100)	99 (43.8)	210 (100)	69 (34.2)	55 (44.0)	28 (31.5)	0	90 (37.2)
Male	627 (41.9)	0	127 (56.2)	0	133 (65.8)	70 (56.0)	61 (68.5)	84 (100)	152 (62.8)
Age at diagnosis (N = 1498)	59 (52; 68)	55 (48; 64)	64 (53; 72)	57 (51; 66)	61 (54; 69)	63 (55; 69)	60 (54; 67)	63 (56; 68)	59 (51; 67)
Time since diagnosis (month) (N = 1307) ^b^	2.4 (1.1; 5.6)	2.0 (0.9; 7.0)	2.8 (1.5; 5.8)	1.9 (1.0; 3.9)	3.0 (1.2; 6.6)	3.8 (1.9; 6.9)	3.1 (2.1; 6.9)	0.4 (0.4; 0.6)	2.6 (1.4; 5.7)
T-stage ^c^ 0/1	295 (19.7)	109 (34.1)	52 (23.0)	56 (26.7)	13 (6.4)	20 (16.0)	21 (23.6)	3 (3.6)	21 (8.7)
2	294 (19.6)	86 (26.9)	38 (16.8)	20 (9.5)	23 (11.4)	26 (20.8)	18 (20.2)	53 (63.1)	30 (12.4)
3	267 (17.8)	20 (6.3)	22 (9.7)	47 (22.4)	85 (42.1)	20 (16.0)	14 (15.7)	21 (25.0)	38 (15.7)
4	163 (10.9)	12 (3.8)	30 (13.3)	1 (0.5)	46 (22.8)	35 (28.0)	23 (25.8)	0	16 (6.6)
Unknown	479 (32.0)	93 (29.1)	84 (37.2)	86 (41.0)	35 (17.3)	24 (19.2)	13 (14.6)	7 (8.3)	137 (56.6)
N-stage 0	476 (31.8)	130 (40.6)	72 (31.9)	55 (26.2)	59 (29.2)	21 (16.8)	31 (34.8)	61 (72.6)	47 (19.4)
1–3	443 (29.6)	74 (23.1)	55 (24.3)	39 (18.6)	95 (47.0)	85 (68.0)	42 (47.2)	12 (14.3)	41 (16.9)
Unknown	579 (38.7)	116 (36.6)	99 (43.8)	116 (55.2)	48 (23.8)	19 (15.2)	16 (18.0)	11 (13.1)	154 (63.6)
Metastasis until baseline No	660 (44.1)	165 (51.6)	129 (57.1)	63 (30.0)	70 (34.7)	25 (20.0)	49 (55.1)	69 (82.1)	90 (37.2)
Yes	435 (29.0)	51 (15.9)	50 (22.1)	51 (24.3)	99 (49.0)	82 (65.6)	17 (19.1)	13 (15.5)	72 (29.8)
Unknown	403 (26.9)	104 (32.5)	47 (20.8)	96 (45.7)	33 (16.3)	18 (14.4)	23 (25.8)	2 (2.4)	80 (33.1)
Disease status Complete remission	371 (24.8)	95 (29.7)	74 (32.7)	41 (19.5)	48 (23.8)	5 (4.0)	17 (19.1)	56 (66.7)	35 (14.5)
Partial remission/ stable	302 (20.2)	68 (21.3)	22 (9.7)	35 (16.7)	54 (26.7)	41 (32.8)	12 (13.5)	13 (15.5)	57 (23.6)
Progress	295 (19.7)	30 (9.4)	37 (16.4)	28 (13.3)	65 (32.2)	36 (28.8)	18 (20.2)	6 (7.1)	75 (31.0)
Unknown	530 (35.4)	127 (39.7)	93 (41.2)	106 (50.5)	35 (17.3)	43 (34.4)	42 (47.2)	9 (10.7)	75 (31.0)
Disease type Primary cancer	1063 (71.0)	230 (71.9)	156 (69.0)	143 (68.1)	142 (68.1)	98 (78.4)	54 (60.7)	73 (86.9)	167 (69.0)
Secondary cancer	122 (8.1)	30 (9.4)	29 (12.8)	12 (5.7)	9 (4.5)	6 (4.8)	5 (5.6)	5 (6.0)	26 (10.7)
Recurrence	201 (13.4)	39 (12.2)	29 (12.8)	41 (19.5)	30 (14.9)	11 (8.8)	23 (25.8)	4 (4.8)	24 (9.9)
Unknown	112 (7.5)	21 (6.6)	12 (5.3)	14 (6.7)	21 (10.4)	10 (8.0)	7 (7.9)	2 (2.4)	25 (10.3)
Psychological comorbidities No	1196 (79.8)	262 (81.9)	196 (86.7)	184 (87.6)	166 (82.2)	73 (58.2)	56 (62.9)	76 (90.5)	183 (75.6)
Yes	222 (14.8)	46 (14.4)	16 (7.1)	23 (11.0)	29 (14.4)	36 (28.8)	29 (32.6)	5 (6.0)	38 (15.7)
Unknown	80 (5.3)	12 (3.8)	14 (6.2)	3 (1.4)	7 (3.5)	16 (12.8)	4 (4.5)	3 (3.6)	21 (8.7)
Physical comorbidities No	565 (37.7)	138 (43.1)	90 (39.8)	100 (47.6)	64 (31.7)	32 (25.6)	39 (43.8)	16 (19.0)	86 (35.5)
Yes	892 (59.5)	177 (55.3)	131 (58.0)	108 (51.4)	127 (62.9)	89 (71.2)	47 (52.8)	67 (79.8)	146 (60.3)
Unknown	41 (2.7)	5 (1.6)	5 (2.2)	2 (1.0)	11 (5.4)	4 (3.2)	3 (3.4)	1 (1.2)	10 (4.1)
Surgery No/planned/ unknown	431 (28.8)	80 (25.0)	33 (14.6)	35 (16.7)	50 (24.8)	99 (79.2)	31 (34.8)	5 (6.0)	98 (40.5)
Yes—treated	1067 (71.2)	240 (75.0)	193 (85.4)	175 (83.3)	152 (75.2)	26 (20.8)	58 (65.2)	79 (94.0)	144 (59.5)
Radiotherapy No/planned/ unknown	1096 (73.2)	214 (66.9)	202 (89.4)	192 (91.4)	131 (64.9)	76 (60.8)	41 (46.1)	77 (91.7)	163 (67.4)
Yes—treated	231 (15.4)	73 (22.8)	16 (7.1)	11 (5.2)	44 (21.8)	35 (28.0)	24 (27.0)	1 (1.2)	27 (11.2)
Yes—in treatment	171 (11.4)	33 (10.3)	8 (3.5)	7 (3.3)	27 (13.4)	14 (11.2)	24 (27.0)	6 (7.1)	52 (21.5)
Chemotherapy No/planned/ unknown	811 (54.1)	154 (48.1)	199 (88.1)	139 (66.2)	80 (39.6)	22 (17.6)	42 (47.2)	76 (90.5)	99 (40.9)
Yes—treated	199 (13.3)	67 (20.9)	11 (4.9)	16 (7.6)	37 (7.6)	23 (18.4)	15 (16.9)	1 (1.2)	29 (12.0)
Yes—in treatment	488 (32.6)	99 (30.9)	16 (7.1)	55 (26.2)	85 (42.1)	80 (64.0)	32 (36.0)	7 (8.3)	114 (47.1)
Stable partnership No	361 (24.1)	84 (26.3)	46 (20.4)	55 (26.2)	49 (24.3)	34 (27.2)	30 (33.7)	7 (8.3)	56 (23.1)
Yes	1070 (71.4)	227 (70.9)	166 (73.5)	146 (69.5)	146 (72.3)	84 (67.2)	56 (62.9)	72 (85.7)	173 (71.5)
Unknown	67 (4.5)	9 (2.8)	14 (6.2)	9 (4.3)	7 (3.5)	7 (5.6)	3 (3.4)	5 (6.0)	13 (5.4)
School Secondary school (up to 9 years)	498 (33.2)	88 (27.5)	88 (38.9)	62 (29.5)	70 (34.7)	61 (48.8)	31 (34.8)	14 (16.7)	84 (34.7)
Secondary school (10 years)	439 (29.3)	113 (35.3)	57 (25.2)	64 (30.5)	61 (30.2)	30 (24.0)	27 (30.3)	22 (26.2)	65 (26.9)
Vocational baccalaureate	142 (9.5)	22 (6.9)	21 (9.3)	24 (11.4)	19 (9.4)	10 (8.0)	10 (11.2)	10 (11.9)	26 (10.7)
Baccalaureate	359 (24.0)	89 (27.8)	51 (22.6)	51 (24.3)	46 (22.8)	17 (13.6)	15 (16.9)	37 (44.0)	53 (21.9)
Unknown	60 (4.0)	8 (2.5)	9 (4.0)	9 (4.3)	6 (3.0)	7 (5.6)	6 (6.7)	1 (1.2)	14 (5.8)
Work Blue collar	250 (16.7)	31 (9.7)	45 (19.9)	21 (10.0)	34 (16.8)	38 (30.4)	31 (34.8)	4 (4.8)	46 (19.0)
White collar	769 (51.3)	195 (60.9)	105 (46.5)	129 (61.4)	106 (52.5)	54 (43.2)	29 (32.6)	45 (53.6)	106 (43.8)
Civil servant	116 (7.7)	30 (9.4)	19 (8.4)	6 (2.9)	16 (7.9)	6 (4.8)	3 (3.4)	13 (15.5)	23 (9.5)
Self-employed	160 (10.7)	22 (6.9)	23 (10.2)	21 (10.0)	22 (10.9)	13 (10.4)	9 (10.1)	16 (19.0)	34 (14.0)
Unknown	203 (13.6)	42 (13.1)	34 (15.0)	33 (15.7)	24 (11.9)	14 (11.2)	17 (19.1)	6 (7.1)	33 (13.6)

^a^ Other: head and neck (N = 31), leukemia and AL-amyloidosis (N = 23), pancreas (N = 36), lymphoma (N = 21), sarcoma (N = 30), myeloma (17), CNS (8), urinary tract (11), CUP (7), other (40), and unknown (4). ^b^ In the model as categorical variable (up to 1 month, 2–4-month, 5–12 month, more than 12 months, and unknown). ^c^ In the model as T0/T1, T2-T4, and Tx.
