# Supplementary material for: 12-Month Trajectories of Health-Related Quality of Life Following Hospitalization in German Cancer Centers—A Secondary Data Analysis
Source: Curr Oncol. 2024 Apr 23;31(5):2376–92. doi: 10.3390/curroncol31050177 (PMC11120277; doi:10.3390/curroncol31050177)
Supplement: Supplementary file 1 [file curroncol-31-00177-s001.zip › curroncol-2935019-supplementary.pdf]

Cancer Type	Timepoint	Mean	95% CI		Differences over time	Differences between groups Reference:						
			lower	upper	t1-t2, t1-t3	Breast	Skin	Gyneco-logical	Digestive tract	Lung	Oral/ facial	Prostate
Breast (C50)	Baseline (t1)	37.19	35.51	38.86	-	-	-	-	-	-	-	-
	6 months (t2)	37.52	35.77	39.27	0.33	-	-	-	-	-	-	-
	12 months (t3)	39.42	37.73	41.11	2.23	-	-	-	-	-	-	-
Skin (C34, C44)	Baseline (t1)	39.72	37.99	41.45	-	2.53	-	-	-	-	-	-
	6 months (t2)	40.56	38.74	42.39	0.84	3.04	-	-	-	-	-	-
	12 months (t3)	40.59	38.67	42.50	0.87	1.17	-	-	-	-	-	-
Gynecological (C51-C58)	Baseline (t1)	32.40	30.53	34.26	-	**-4.79**	**-7.32**	-	-	-	-	-
	6 months (t2)	36.37	34.26	38.48	3.97	-1.15	**-4.19**	-	-	-	-	-
	12 months (t3)	37.10	34.95	39.26	**4.70**	-2.32	-3.49	-	-	-	-	-
Digestive tract (C16-C21)	Baseline (t1)	36.12	34.33	37.91	-	-1.07	**-3.60**	**3.72**	-	-	-	-
	6 months (t2)	38.11	36.25	39.98	1.99	0.59	-2.45	1.74	-	-	-	-
	12 months (t3)	38.63	36.65	40.61	2.51	-0.79	-1.96	1.53	-	-	-	-
Lung (C34)	Baseline (t1)	33.90	31.94	35.86	-	-3.29	**-5.82**	1.5	-2.22	-	-	-
	6 months (t2)	34.31	32.03	36.59	0.41	-3.21	**-6.25**	-2.06	-3.8	-	-	-
	12 months (t3)	33.74	31.22	36.26	-0.16	**-5.68**	**-6.85**	-3.36	**-4.89**	-	-	-
Oral/facial (C01-C14)	Baseline (t1)	35.93	33.68	38.18	-	-1.26	-3.79	3.53	-0.19	2.03	-	-
	6 months (t2)	36.72	34.13	39.30	0.79	-0.80	-3.84	0.35	-1.39	2.41	-	-
	12 months (t3)	38.35	35.53	41.17	2.42	-1.07	-2.24	1.25	-0.28	4.61	-	-
Prostate/germ cell (C61, C62)	Baseline (t1)	34.97	32.27	37.66	-	-2.22	**4.75**	2.57	-1.15	1.07	-0.96	-
	6 months (t2)	39.55	36.70	42.4	4.58	2.03	1.01	3.18	1.44	**5.24**	2.83	-
	12 months (t3)	39.61	36.75	42.48	4.64	0.19	0.98	2.51	0.98	**5.87**	1.26	-
Other	Baseline (t1)	36.26	34.61	37.91	-	-0.93	**3.46**	**3.86**	0.14	2.36	0.33	1.29
	6 months (t2)	37.42	35.41	39.44	1.16	-0.10	3.14	1.05	-0.69	3.11	0.7	-2.13
	12 months (t3)	38.32	36.28	40.35	2.06	-1.10	2.27	1.22	-0.31	**4.58**	-0.03	-1.29

Table S1: Trajectories (values) of HRQoL-scores (PCS) after hospitalisation at baseline. PCS= SF 12 Physical Health Summary Scale. Values adjusted for: age at diagnosis, sex, time of survey, time since diagnosis, metastatic disease, T-stage, N-stage, disease status, reason for hospitalisation, physical comorbidities, emotional comorbidities, radiotherapy, chemotherapy, surgery, education, partnership, occupation, interactions: time of survey by cancer type and time since diagnosis by cancer type. Bold: Significant differences. 95% CI: 95% confidence interval.

Cancer Type	Timepoint	Mean	95% CI		Differences over time	Differences between groups Reference:						
			lower	upper	t1-t2, t1-t3	Breast	Skin	Gyneco-logical	Digestive tract	Lung	Oral/ facial	Prostate
Breast (C50)	Baseline (t1)	42.17	40.06	44.28	-	-	-	-	-	-	-	-
	6 months (t2)	44.97	42.79	47.14	2.80	-	-	-	-	-	-	-
	12 months (t3)	44.98	42.76	47.2	2.81	-	-	-	-	-	-	-
Skin (C34, C44)	Baseline (t1)	43.80	41.50	46.11	-	1.63	-	-	-	-	-	-
	6 months (t2)	44.99	42.69	47.28	1.19	0.02	-	-	-	-	-	-
	12 months (t3)	45.01	42.60	47.42	1.21	0.03	-	-	-	-	-	-
Gynecological (C51-C58)	Baseline (t1)	42.93	40.54	45.32	-	0.76	-0.87	-	-	-	-	-
	6 months (t2)	44.04	41.30	46.78	1.11	-0.93	-0.95	-	-	-	-	-
	12 months (t3)	46.07	43.42	48.73	3.14	1.09	1.06	-	-	-	-	-
Digestive tract (C16-C21)	Baseline (t1)	43.57	41.37	45.77	-	1.40	-0.23	0.64	-	-	-	-
	6 months (t2)	43.38	40.91	45.84	-0.19	-1.59	-1.61	-0.66	-	-	-	-
	12 months (t3)	43.98	41.44	46.52	0.41	-1.00	-1.03	-2.09	-	-	-	-
Lung (C34)	Baseline (t1)	43.39	40.79	45.99	-	1.22	-0.41	0.46	-0.18	-	-	-
	6 months (t2)	42.22	39.04	45.41	-1.17	-2.75	-2.77	-1.82	-1.16	-	-	-
	12 months (t3)	40.94	37.58	44.30	-2.45	-4.04	-4.07	-5.13	-3.04	-	-	-
Oral/facial (C01-C14)	Baseline (t1)	42.43	39.63	45.23	-	0.26	-1.37	-0.5	-1.14	-0.96	-	-
	6 months (t2)	43.63	40.33	46.94	1.20	-1.34	-1.36	-0.41	0.25	1.41	-	-
	12 months (t3)	44.08	40.73	47.43	1.65	-0.90	-0.93	-1.99	0.1	3.14	-	-
Prostate/germ cell (C61, C62)	Baseline (t1)	40.84	35.99	45.68	-	-1.33	-2.96	-2.09	-2.73	-2.55	-1.59	-
	6 months (t2)	41.33	36.39	46.28	0.49	-3.64	-3.66	-2.71	-2.05	-0.89	-2.3	-
	12 months (t3)	43.15	38.26	48.03	2.31	-1.83	-1.86	-2.92	-0.83	2.21	-0.93	-
Other	Baseline (t1)	43.19	41.21	45.18	-	1.02	-0.61	0.26	-0.38	-0.20	0.76	2.35
	6 months (t2)	43.73	41.31	46.15	0.54	-1.24	-1.26	-0.31	0.35	1.51	0.1	2.4
	12 months (t3)	45.20	42.77	47.63	2.01	0.22	0.19	-0.87	1.22	4.26	1.12	2.05

Table S2: Trajectories (values) of HRQoL-scores (MCS) after hospitalisation at baseline. MCS= SF 12 Mental Health Summary Scale. Values adjusted for: age at diagnosis, sex, time of survey, time since diagnosis, metastatic disease, T-stage, N-stage, disease status, reason for hospitalisation, physical comorbidities, emotional comorbidities, radiotherapy, chemotherapy, surgery, education, partnership, occupation, interactions: time of survey by cancer type and time since diagnosis by cancer type. Bold: Significant differences. 95% CI: 95% confidence interval.

Results of the Generalized Linear Model. Interaction terms

		Modell PCS			Modell MCS		
Variable	Value	B	95% CI	p	B	95% CI	p
Time point*cancer type							
	t1 x breast (ref)						
	t1 * skin	0.51	-1.07; 2.09	0.53	-1.61	-3.51; 0.29	0.10
	t1 * gynecological	3.64	1.73; 5.55	<0.01	-1.69	-3.83; 0.46	0.12
	t1 * digestive tract	1.66	-0.13; 3.44	0.07	-2.99	-5.26; -0.72	0.01
	t1 * lung	0.08	-2.08; 2.23	0.95	-3.96	-6.72; -1.20	0.01
	t1 * oral/facial	0.45	-2.12; 3.01	0.73	-1.59	-4.43; 1.25	0.27
	t1 * prostate/germ cell	4.25	2.14; 6.35	<0.01	-2.30	-4.58; -0.03	<0.05
	t1 * other	0.83	-1.07; 2.72	0.39	-2.26	-4.50; -0.01	<0.05
	t2 * breast						
	t2 * skin	-1.36	-2.93; 0.20	0.09	-1.60	-3.63; 0.43	0.12
	t2 * gynecological	2.47	0.57; 4.38	0.01	0.33	-1.87; 2.53	0.77
	t2 * digestive tract	0.28	-1.54; 2.10	0.76	-2.40	-4.71; -0.08	0.04
	t2 * lung	-2.39	-4.73; -0.05	<0.05	-5.25	-8.27; -2.24	<0.01
	t2 * oral/facial	0.19	-2.52; 2.89	0.89	-1.16	-3.97; 1.66	0.42
	t2 * prostate/germ cell	2.42	0.36; 4.47	0.02	-0.50	-2.89; 1.89	0.68
	t2 * other	-0.18	-2.10; 1.75	0.86	-0.80	-3.09; 1.49	0.49
Time since diagnosis*cancer type							
	skin * 0/1 month						
	skin * 2-4 months	0.04	-3.44; 3.51	0.98	1.64	-3.07; 6.35	0.49
	skin * 5-12 months	-3.60	-7.76; 0.57	0.09	-0.41	-6.05; 5.22	0.89
	skin * >12 months	0.38	-4.46; 5.22	0.88	1.75	-4.41; 7.91	0.58
	skin * unknown	1.08	-3.58; 5.74	0.65	0.55	-5.19; 6.30	0.85
	gynecological * 0/1 month						
	gynecological * 2-4 months	-0.94	-4.11; 2.24	0.56	-0.23	-4.36; 3.91	0.91
	gynecological * 5-12 months	-2.11	-5.92; 1.70	0.28	0.66	-4.79; 6.11	0.81
	gynecological * >12 months	-1.89	-7.19; 3.41	0.48	-2.41	-8.22; 3.40	0.42
	gynecological * unknown	-5.81	-10.81; -0.80	0.02	0.07	-6.34; 6.48	0.98
	digestive tract * 0/1 month						
	digestive tract * 2-4 months	-2.19	-5.66; 1.28	0.22	0.12	-4.42; 4.65	0.96
	digestive tract * 5-12 months	-2.37	-6.64; 1.90	0.28	0.07	-5.30; 5.44	0.98
	digestive tract * >12 months	-1.32	-5.96; 3.32	0.58	1.23	-4.43; 6.89	0.67
	digestive tract * unknown	-2.30	-7.67; 3.07	0.40	-0.07	-5.59; 5.46	0.98
	lung * 0/1 month						
	lung * 2-4 months	1.31	-3.53; 6.15	0.60	-0.77	-7.56; 6.03	0.83
	lung * 5-12 months	0.24	-4.68; 5.17	0.92	-0.50	-7.43; 6.42	0.89
	lung * >12 months	3.24	-3.14; 9.62	0.32	1.76	-6.17; 9.69	0.66
	lung * unknown	-0.17	-6.20; 5.85	0.96	2.51	-5.77; 10.79	0.55
	oral/facial * 0/1 month						
	oral/facial * 2-4 months	1.87	-4.07; 7.81	0.54	5.74	-2.76; 14.23	0.19
	oral/facial * 5-12 months	-0.59	-7.30; 6.12	0.86	4.15	-5.17; 13.48	0.38
	oral/facial * >12 months	1.69	-6.28; 9.65	0.68	8.56	-0.30; 17.41	0.06
	oral/facial * unknown	-1.66	-8.58; 5.27	0.64	3.58	-6.22; 13.37	0.47
	prostate/germ cell * 0/1 month						
	prostate/germ cell * 2-4 months	-4.74	-10.58; 1.12	0.11	-4.11	-14.01; 5.79	0.42
	prostate/germ cell * 5-12 months	-7.90	-14.63; -1.17	0.02	0.21	-7.91; 8.32	0.96
	prostate/germ cell * >12 months	-9.28	-18.40; -0.17	<0.05	-17.38	-36.87; 2.11	0.08
	prostate/germ cell * unknown	-2.82	-7.28; 1.65	0.22	-4.53	-10.22; 1.17	0.12
	other * 0/1 month						
	other * 2-4 months	-1.91	-5.21; 1.39	0.26	-3.81	-8.22; 0.61	0.09
	other * 5-12 months	-0.98	-4.85; 2.89	0.62	-4.17	-9.22; 0.89	0.11
	other * >12 months	0.69	-4.40; 5.78	0.79	-1.11	-7.27; 5.05	0.72
	other * unknown	-2.85	-7.32; 1.63	0.21	-5.34	-10.71; 0.02	0.05

Table S3: Results of the Generalized Linear Model. Interaction terms (all other results in table 3). PCS= SF12 Physical Health Summary Scale, MCS= SF12 Mental Health Summary Scale, B= non-standardized regression coefficient (indicating a B point increase or decrease in the respective scale); 95% CI: 95% confidence interval; p = p-value.
